# Persistent Type I Endoleak after Endovascular Treatment with Chimney Technique

**DOI:** 10.3389/fcvm.2016.00032

**Published:** 2016-09-20

**Authors:** Ana Isabel Azevedo, Pedro Braga, Alberto Rodrigues, Nuno Ferreira, Marlene Fonseca, Adelaide Dias, Vasco Gama Ribeiro

**Affiliations:** ^1^Department of Cardiology, Vila Nova de Gaia/Espinho Hospital Center, Vila Nova de Gaia, Portugal

**Keywords:** endovascular aortic repair, aortic dissection, endovascular intervention, vascular complications, endoleak

## Abstract

Thoracic endovascular aortic repair (TEVAR) is increasingly used in the treatment of acute type B aortic dissection. Type Ia endoleaks are a common complication of the procedure, but its clinical significance and the best treatment strategy remain poorly defined. We present a case of a type Ia endoleak following TEVAR in the treatment of acute type B aortic dissection. Chimney technique approach was used in an attempt to seal the endoleak. Although technical success was suboptimal, the patient remained clinically stable and event free. Data regarding the natural course and management of type Ia endoleaks following TEVAR for aortic dissection are sparse. Future research is required to establish the clinical and technical determinants of the need to treat these endoleaks and the best treatment strategy.

## Introduction

Aortic dissection has one of the highest mortality rates of cardiovascular diseases, and its management remains a challenge. Acute complicated type B aortic dissection may present with signs of organ malperfusion, uncontrolled hypertension, or signs of impending rupture such as an increase in periaortic hematoma and hemorrhagic pleural effusion in two subsequent computed tomography (CT) examinations. Thoracic endovascular aortic repair (TEVAR) has shown a survival benefit when compared with open surgery and should be considered as first-line treatment of acute type B aortic dissection (1). Type Ia endoleak is a possible complication of TEVAR and is due to an inadequate seal at the proximal end of the endograft (2). Type Ia endoleaks immediately following TEVAR in patients with type B aortic dissection are relatively common, and their immediate appearance can be an indication of worse aortic condition (3). Chimney technique represents a viable treatment option in patients with challenging aortic arch pathology by prolonging the proximal landing zone while maintaining aortic side branches perfusion (4).

## Background

### Clinical History

**Figure 1:**
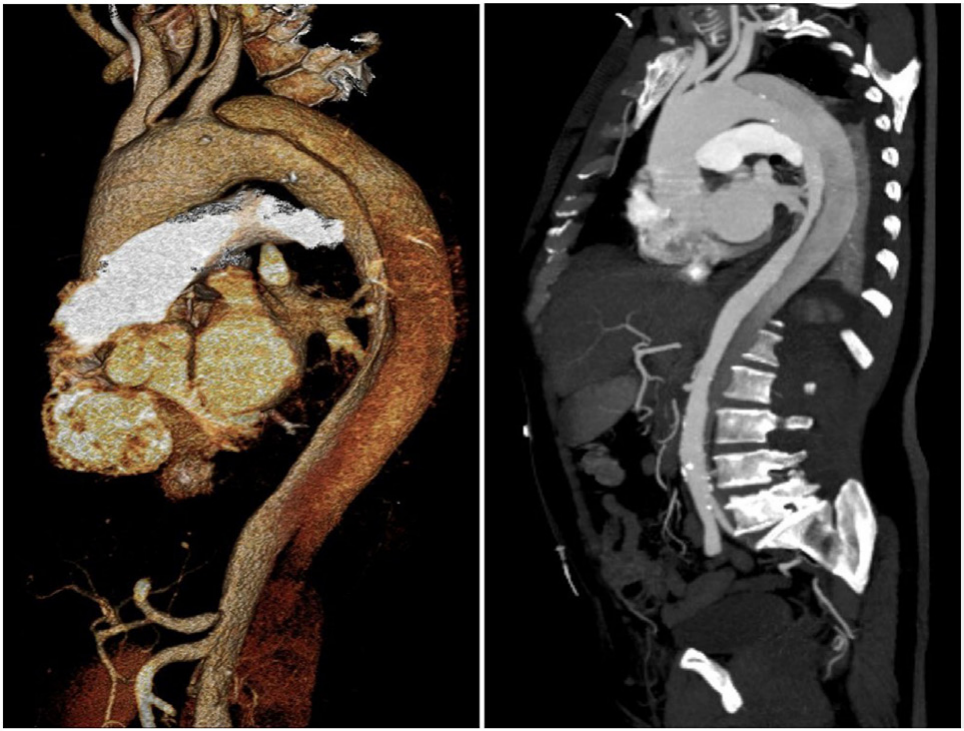
**AngioCT showing Stanford type B aortic dissection**.

**Figure 2:**
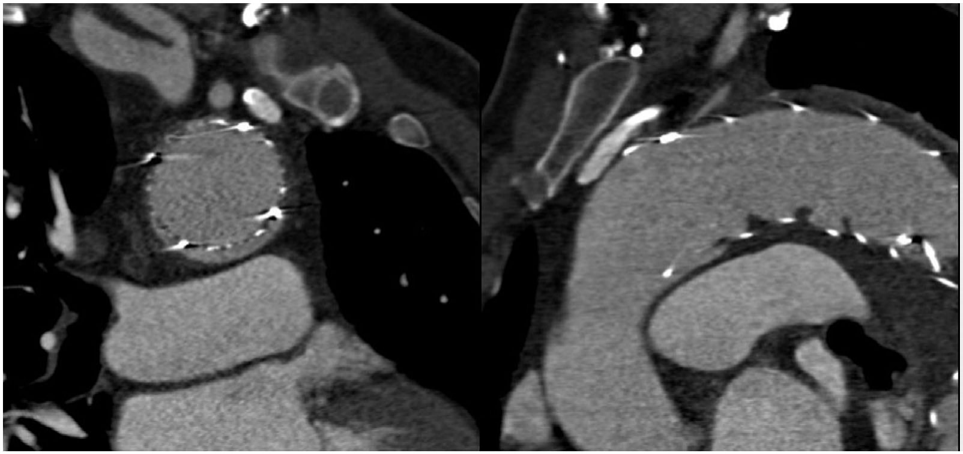
**AngioCT showing type Ia endoleak after the TEVAR procedure**.

A 65-year-old man, with history of uncontrolled hypertension, presented at our Center with acute thoracic and back pain. AngioCT revealed an acute Stanford type B aortic dissection, extending from the proximal descending thoracic aorta (distal to the origin of the left subclavian artery) to the iliac arteries (Figure 1). Aortic sizing was performed using CT, and aortic diameter proximal to the origin of the left subclavian artery was 41 mm. Medical therapy in order to control blood pressure was optimized. Despite four different classes of antihypertensive drugs, including a diuretic, at maximal tolerated doses, blood pressure values were persistently high. Thus, a case of an acute complicated (refractory hypertension) type B aortic dissection was presented and discussed by the Heart Team (including General and Interventional Cardiologists, Cardiothoracic Surgeons, Anesthesiologists, and experts in Cardiovascular Imaging), and a TEVAR was considered to be in the best interest of this patient. Implantation of an aortic prosthesis (Valiant^®^ Captivia^®^ 42 mm × 150 mm) with planned left subclavian artery coverage in order to cover the primary entry tear in the proximal thoracic descending aorta was performed. After the procedure, the patient was asymptomatic. AngioCT after the procedure showed occlusion of the left subclavian artery and an endoleak at the proximal graft attachment site – type Ia endoleak (Figure 2). The patient was discharged with optimized antihypertensive drug therapy and remained under close follow-up.

### Follow-up

A year later, the patient complained about lower back pain. AngioTC showed persistency of the aortic dissection to the iliac arteries and no increase in the size of the type Ia endoleak. Since the recurrence of symptoms is one of the signs of instability in the chronic phase of aortic dissection, we decided to perform a second TEVAR, placing an aortic endoprosthesis (Valiant^®^ 46 mm × 150 mm) at the level of the descending aortic, partially covering the distal part of the first endoprosthesis.

The patient remained clinically stable, and clinical and imagiological follow-up was performed.

**Figure 3:**
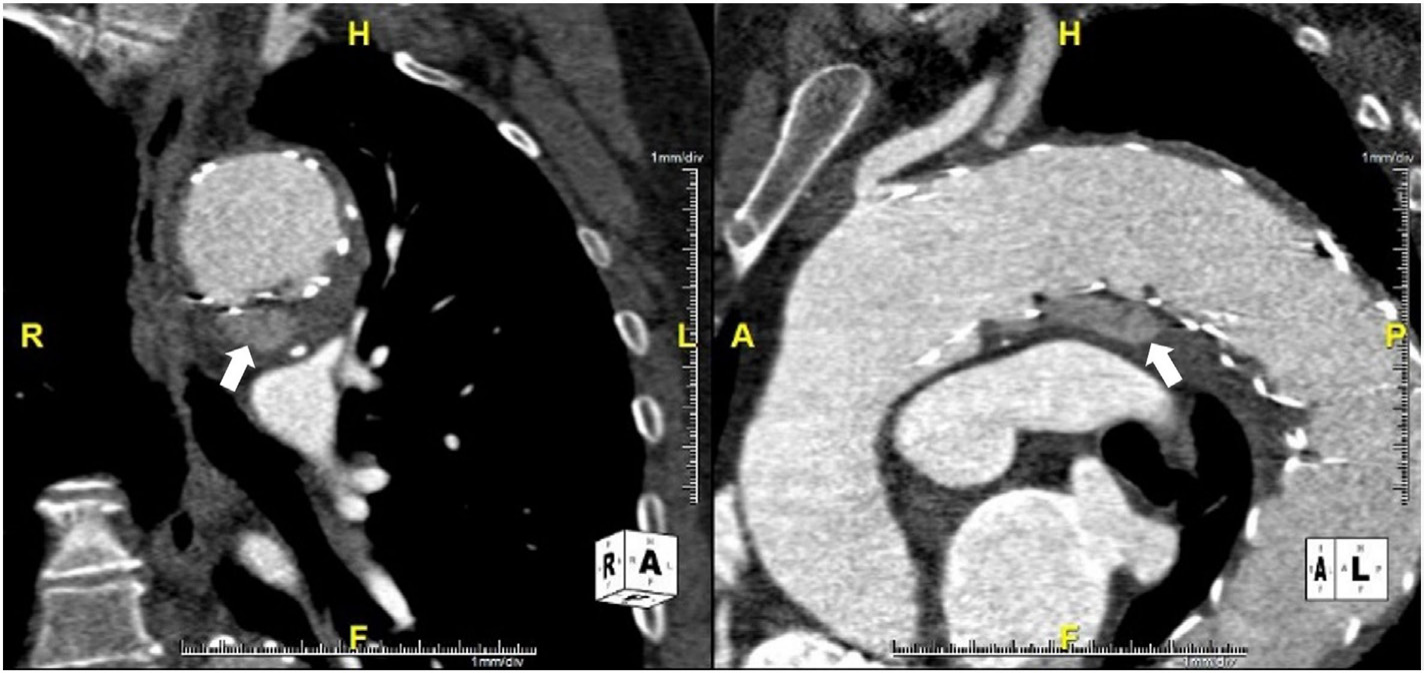
**AngioCT showing the increase in type Ia endoleak dimension (arrows)**.

Two years after the first procedure, the patient complained about thoracic discomfort. AngioCT showed an increase in endoleak dimension at the level of the transition between the aortic arch and the proximal descending aorta (Figure 3).

The case was re-evaluated by the Heart Team, and we decided to treat the endoleak. In order to do so, we faced the need of maintaining perfusion in the supra-aortic branches. Several treatment options were discussed: placement of a coil at the site of the endoleak would be a less complex procedure, but the entry site was subject to high flow pressure, and it was considered not to be the best option; a fenestrated prosthesis could resolve the problem of the side branches perfusion, but the prosthesis was not readily available, and it would take up to several weeks in order to achieve it; and a surgical approach was considered to be of high risk.

**Figure 4:**
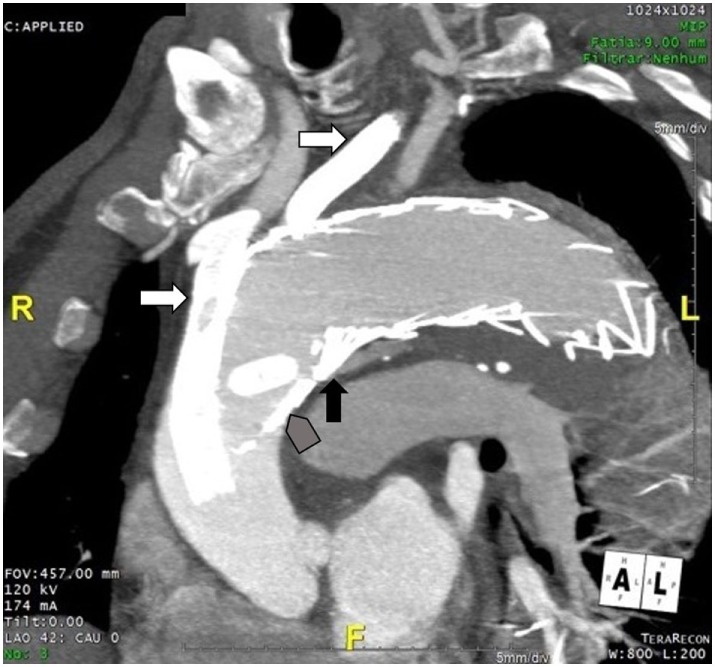
**Final position of the prosthesis**. White arrows: self-expandable stents at the brachiocephalic trunk and left common carotid artery, placed between aortic wall and aortic endoprosthesis. Black arrow: proximal entry site of the endoleak. Arrow head: proximal landing zone of the aortic endoprosthesis.

Taking this into account and based on our previous experience with the Chimney technique, we decided do use this approach. A detailed CT review and evaluation of the aortic arch and supra-aortic branches configuration and dimension was undertaken. Proximal landing zone of the endoprosthesis was set proximal to the origin of the brachiocephalic trunk (dimension 43 mm × 47 mm), covering the brachiocephalic trunk and left common carotid artery, which would be then perfused by deployment of self-expandable stents, placed between the endoprosthesis and the aortic wall (Figure 4).

Surgical access to the left common carotid artery and percutaneous access through right axillary artery to the brachiocephalic trunk and through the left femoral artery to the aorta were planned. After placement of the guidewires into the supra-aortic branches, a third aortic endoprosthesis (GORE^®^TAG^®^ 45 mm × 150 mm) was placed at the level of the aortic arch, proximal to the first one and covering the aortic branches, in order to seal the endoleak. To ensure the patency of the side branches, self-expandable stents (GORE^®^VIABAHN^®^) were deployed in the brachiocephalic trunk and the left common carotid artery (in Supplementary Material Video 1). Despite the attempted correction, the type Ia endoleak persisted, with only a small decrease in its dimension (in Supplementary Material Video 2). The flow at the vertebral artery was evaluated by CT before and after the procedure, and no abnormalities were found.

Previous to the intervention, the patient was on single antiplatelet therapy (aspirin 150 mg daily), an ACE-inhibitor, a beta-blocker, a calcium channel blocker, a diuretic, and a statin. After the intervention, dual antiplatelet therapy was initiated (aspirin 150 mg daily plus clopidogrel 75 mg daily) and the remaining therapy maintained.

**Figure 5:**
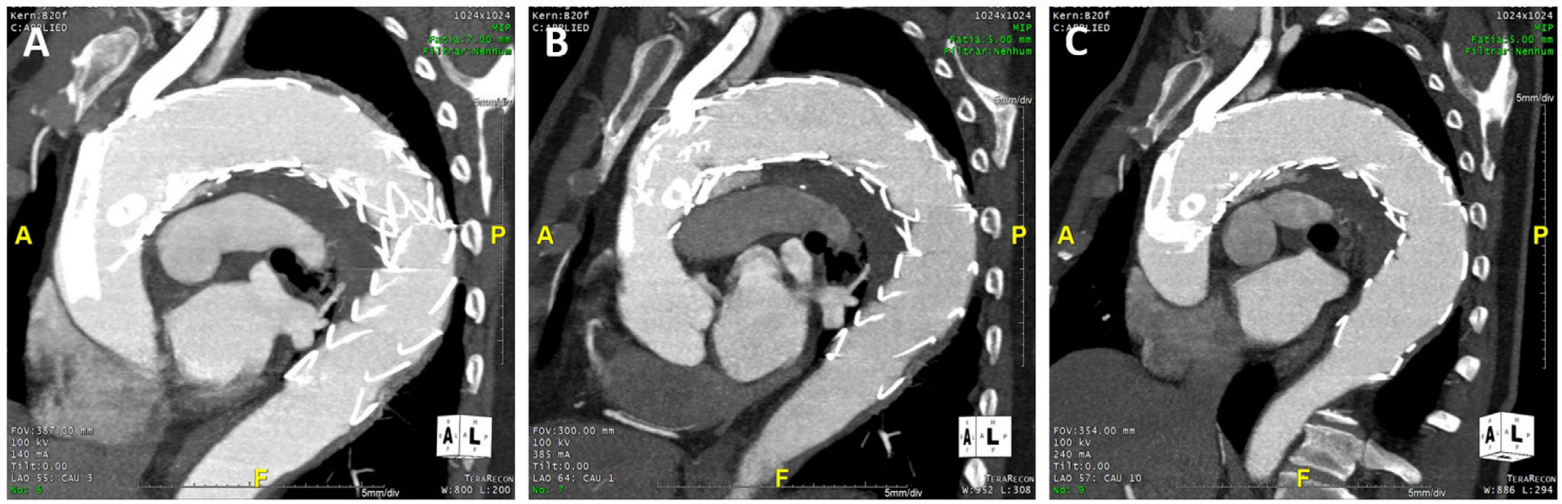
**AngioCT at 1 month (A), 3 months (B), and 7 months follow-up (C)**.

The patient remained asymptomatic. AngioCT at 1, 3, and 7 months follow-up (Figures 5A–C, respectively) confirmed patency of the stents and the persistency of the endoleak, with no increase in its dimension.

## Discussion

### Type Ia Endoleaks Following TEVAR for Aortic Dissection

The etiology and pathology of aortic dissection is distinct from aortic aneurysms. Type Ia endoleak following TEVAR for thoracic aortic aneurysms is considered a procedure failure, and aggressive treatment is recommended to eliminate the leakage. On the other hand, a type Ia endoleak after TEVAR for the treatment of aortic dissection does not result in systemic pressurization of the cul-de sac and might not place the patient at the risk of rupture (3). Type Ia endoleak may result from the angulation or tortuosity of the aortic arch, and a short proximal landing zone and its immediate appearance seems to be an indication of worse aortic condition (3). One of the main issues in addressing aortic arch pathologies is the short proximal landing zone, since an adequate length has to be present to ensure successful proximal fixation (4). Chimney technique, also described as periscope grafts technique, involves the placement of stents in side branches of aorta alongside the main endovascular stent graft and has shown to be a viable option in the treatment of thoracoabdominal aortic aneurysms and in aortic arch pathology, but its use in the treatment of type Ia endoleaks following TEVAR for aortic dissection is still limited (5, 6).

### Treatment Options

The presence of an endoleak following TEVAR for the treatment of type B aortic dissection may not adversely impact technical success and short-term outcomes, as opposed to the treatment of aortic aneurysms. In fact, hemodynamic changes in the aorta may result in the sealing of the entry site and filling of the space with thrombotic material, resulting in the spontaneous disappearance of the endoleak. However, the immediate appearance of an endoleak seems to point to a worse aortic condition and less likelihood of spontaneous resolution (3).

Several endovascular treatment options for type Ia endoleaks have been applied, mainly after (T)EVAR for thoracic or abdominal aortic aneurysm repair.

Insertion of an aortic cuff to extend endograft coverage more proximally or the placement of a large-caliber balloon expandable stent inside the proximal endograft to promote the seal is one of the treatment options available. As type Ia endoleaks frequently result from an angulated and tortuous aortic arch and short proximal landing zones, this treatment option may not be sufficient to promote the seal.

The use of customized fenestrated or branched endografts has shown promising results in aortic arch aneurysms. However, they are complex, technically challenging, and expensive procedures, and the material may not be immediately available for urgent procedures.

The Chimney (landing zones 1 and 2) or the sandwich technique (landing zone 0), in which standard aortic endografts and simultaneous branch vessel stenting are used, are increasingly more common alternative procedures for the treatment of aortic pathology extending to the aortic arch, including aortic dissection, aneurysm, rupture, or proximal endoleaks. The advantages of this technique include readily available (“on-the-shelf”) stents, placed in aortic side branches, creating a prolonged proximal aortic landing zone without compromising the perfusion of the branches (7, 8). In the initial approach of this patient, although we recognized there was a short proximal landing zone, we considered that performing a TEVAR with planned left subclavian artery coverage would be a more simple procedure and sufficient to seal the proximal entry site. As the endoleak appeared, Chimney technique was later used to try to resolve it, by achieving proximal coverage.

Another treatment option available for patients not eligible for these interventions due to severe comorbidities or anatomical factors is transcatheter embolization of the leak. Previous published experiences include endoleak embolization using coils, cyanoacrylate, and Onyx™, a relatively novel liquid embolic agent, most commonly used to treat intracranial arteriovenous malformations (9–11). A recent fixation system (Heli-FX EndoAnchor System, Aptus Endosystems, Sunnyvale, CA, USA) has successfully resolve type Ia endoleaks and endograft migration in patients with thoracic or abdominal aortic aneurysms that underwent (T)EVAR (12). There is still limited published experience of type I endoleaks embolization, and the vast majority of the cases are of endoleaks following endovascular treatment of thoracic and abdominal aortic aneurysms and not aortic dissections.

The option of conventional open surgery allows easy access the site of the endoleak but carries particular high risk in middle- and old-aged, frail patients, with several comorbidities, who represent the majority of the population with aortic disease.

We decided to use the Chimney technique because of our previous experience with this procedure and local availability of material. At short- and mid-term follow-up, patency of the stents was preserved, but there was only a small decrease in the size of the endoleak. In future cases, a strategy of “watchful waiting” is to be considered, because long-term outcomes of the persistent type Ia endoleak in a dissected aorta remain undefined. If a treatment option is to be chosen, the use of a fenestrated prosthesis could be advantageous. The success of this technique in the treatment of type Ia endoleaks after TEVAR for aortic aneurysms has been described as reasonable, with no secondary rupture and high target vessel patency (5). However, its results in the treatment of type Ia endoleak following TEVAR for aortic dissection repair are sparse. In either case, long-term clinical and CT follow-up remain essential.

## Concluding Remarks

Type Ia endoleak is a known and common complication of endovascular treatment of type B aortic dissection. Chimney technique represents a treatment option in patients with type Ia endoleak at the level of the aortic arch, but despite the efforts undertaken to treat it, technical success can be difficult to achieve. Although the type Ia endoleak persisted, the patient remained clinically stable. Therefore, although the intervention was technically unsuccessful, there was a clinical improvement.

In future similar cases, the risk of a persistent endoleak must be taken into account by the Heart Team when selecting the most appropriate treatment strategy. Furthermore, the patient should be fully informed about the possible complications of the procedure and involved in the treatment decision.

Clinical and imagological (angioCT) follow-up are mandatory, although the clinical significance and prognostic implications of a persistent type Ia endoleak following endovascular treatment of aortic dissection remain poorly defined. In future research, clinical and technical determinants of the need of type Ia endoleak closure and the best treatment option need to be addressed.

## Ethics Statement

Protection of human and animal subjects: the authors declare that no experiments were performed on humans or animals for this study. Confidentiality of data: the authors declare that no patient data are showed in this article. Written informed consent was obtained.

## Author Contributions

All authors gave the following contributions: (1) substantial contributions to the conception/design of the work or the acquisition, analysis, or interpretation of data for the work; (2) drafting the work or revising it critically for important intellectual content; (3) final approval of the version to be published; and (4) agreement to be accountable for all aspects of the work in ensuring that questions related to the accuracy or integrity of any part of the work are appropriately investigated and resolved.

## Conflict of Interest Statement

The authors declare that the research was conducted in the absence of any commercial or financial relationships that could be construed as a potential conflict of interest.

## References

[1] Fattori R Cao P De Rango P Czerny M Evangelista A Nienaber C Interdisciplinary expert consensus document on management of type B aortic dissection. J Am Coll Cardiol (2013) 61:1661–78. 10.1016/j.jacc.2012.11.072 23500232

[2] Millen AM Osman K Antoniou GA McWilliams RG Brennan JA Fisher RK. Outcomes of persistent intraoperative type Ia endoleak after standard endovascular aneurysm repair. J Vasc Surg (2015) 61:1185–91. 10.1016/j.jvs.2014.12.041 25656591

[3] Huang W Yang F Luo J Xie N He P Luo S Outcomes and morphologic changes of immediate type Ia endoleak following endovascular repair of acute type B aortic dissection. Ann Vasc Surg (2014) 29:174–82. 10.1016/j.avsg.2014.10.015 25463345

[4] Hogendoorn W Schlösser FJV Moll FL Sumpio BE Muhs BE Thoracic endovascular aortic repair with chimney graft technique. J Vasc Surg (2013) 58:502–11. 10.1016/j.jvs.2013.03.043 23697513

[5] Montelione N Pecoraro F Puippe G Chaykovska L Rancic Z Pfammatter T A 12-year experience with chimney and periscope grafts for treatment of type I endoleaks. J Endovasc Ther (2015) 22(4):568–74. 10.1177/1526602815586972 25969150

[6] Lobato AC Cury M Pereira FLC Ribeiro VG Camacho-Lobato L Is pure endovascular treatment the new frontier? Endovasc Today (2014):77–81. Available at: http://evtoday.com/2014/11/is-pure-endovascular-treatment-the-new-frontier/

[7] Cires G Noll RE Jr Albuquerque FC Jr Tonnessen BH Sternbergh WC 3rd. Endovascular debranching of the aortic arch during thoracic endograft repair. J Vasc Surg (2011) 53:1485–91. 10.1016/j.jvs.2011.01.053 21498028

[8] Kawatani Y Hayashi Y Ito Y Kurobe H Nakamura Y Suda Y A case of ruptured aortic arch aneurysm successfully treated by thoracic endovascular aneurysm repair with chimney graft. Case Rep Surg (2015) 2015:780147. 10.1155/2015/780147 25815238 PMC4359878

[9] Schiattarella GG Magliulo F Laurino FI Bottino R Bruno AG De Paulis M Transradial approach for the endovascular treatment of type I endoleak after aortic aneurysm repair: a case report. BMC Surg (2013) 13(Suppl 2):S47. 10.1186/1471-2482-13-S2-S47 24267381 PMC3851154

[10] Bangard C Franke M Pfister R Deppe AC Matoussevitch V Maintz D Thoracic type Ia endoleak: direct percutaneous coil embolization of the aortic arch at the blood entry site after TEVAR and double-chimney stent-grafts. Eur Radiol (2014) 24:1430–4. 10.1007/s00330-014-3143-8 24643498

[11] Buckenham T McKewen M Laing A Roake J Lewis D Gordon MK. Cyanoacrylate embolization of endoleaks after abdominal aortic aneurysm repair. ANZ J Surg (2009) 79:841–3. 10.1111/j.1445-2197.2009.05113.x 20078537

[12] Jordan WD Jr Mehta M Varnagy D Moore WM Jr Arko FR Joye J Results of the ANCHOR prospective, multicenter registry of EndoAnchors for type Ia endoleaks and endograft migration in patients with challenging anatomy. J Vasc Surg (2014) 60:885–92. 10.1016/j.jvs.2014.04.063 25088739

